# Air-pollutant chemicals and oxidized lipids exhibit genome-wide synergistic effects on endothelial cells

**DOI:** 10.1186/gb-2007-8-7-r149

**Published:** 2007-07-26

**Authors:** Ke Wei Gong, Wei Zhao, Ning Li, Berenice Barajas, Michael Kleinman, Constantinos Sioutas, Steve Horvath, Aldons J Lusis, Andre Nel, Jesus A Araujo

**Affiliations:** 1 Department of Medicine, David Geffen School of Medicine, University of California, Los Angeles, CA 90095, USA; 2 Departments of Human Genetics and Biostatistics, University of California, Los Angeles, CA 90095, USA; 3 Department of Community and Environmental Medicine, University of California, Irvine, CA 92697, USA; 4 Department of Civil and Environmental Engineering, University of Southern California, Los Angeles, CA 90089, USA

## Abstract

**Background:**

Ambient air pollution is associated with increased cardiovascular morbidity and mortality. We have found that exposure to ambient ultrafine particulate matter, highly enriched in redox cycling organic chemicals, promotes atherosclerosis in mice. We hypothesize that these pro-oxidative chemicals could synergize with oxidized lipid components generated in low-density lipoprotein particles to enhance vascular inflammation and atherosclerosis.

**Results:**

We have used human microvascular endothelial cells (HMEC) to study the combined effects of a model air pollutant, diesel exhaust particles (DEP), and oxidized 1-palmitoyl-2-arachidonyl-*sn*-glycero-3-phosphorylcholine (ox-PAPC) on genome-wide gene expression. We treated the cells in triplicate wells with an organic DEP extract, ox-PAPC at various concentrations, or combinations of both for 4 hours. Gene-expression profiling showed that both the DEP extract and ox-PAPC co-regulated a large number of genes. Using network analysis to identify coexpressed gene modules, we found three modules that were most highly enriched in genes that were differentially regulated by the stimuli. These modules were also enriched in synergistically co-regulated genes and pathways relevant to vascular inflammation. We validated this synergy *in vivo *by demonstrating that hypercholesterolemic mice exposed to ambient ultrafine particles exhibited significant upregulation of the module genes in the liver.

**Conclusion:**

Diesel exhaust particles and oxidized phospholipids synergistically affect the expression profile of several gene modules that correspond to pathways relevant to vascular inflammatory processes such as atherosclerosis.

## Abstract 2

Gene expression analysis of human microvascular endothelial cells exposed to diesel exhaust particles and oxidized phospholipids revealed several upregulated gene modules, including genes involved in vascular inflammatory processes such as atherosclerosis.

## Background

Atherosclerotic cardiovascular disease is the leading cause of death in the Western world. In addition to the classical risk factors such as serum lipids, smoking, hypertension, aging, gender, family history, physical inactivity, and diet, recent data have implicated air pollution as an important additional risk factor for atherosclerosis [1]. The strongest and most consistent association between air pollution and cardiovascular morbidity and mortality has been ascribed to ambient particulate matter (PM) [2-6]. Large-scale prospective epidemiological studies have shown that residence in areas with high ambient PM levels is associated with an increased risk of premature cardiopulmonary death [7]. A study by the American Cancer Society reported a 6% increase in cardiopulmonary deaths for every elevation of 10 μg/m^3 ^in PM concentration [8]. Although the mechanism of cardiovascular injury by PM is poorly understood, it has been shown that the particles are coated by a number of chemical compounds, including organic hydrocarbons (for example, polycyclic aromatic hydrocarbons and quinones), transition metals, sulfates and nitrates. In studies looking at the effects of diesel exhaust particles (DEP) on the lung, we and others have shown that the redox cycling organic hydrocarbons and transition metals are capable of generating airway inflammation through their ability to generate reactive oxygen species (ROS) and oxidative stress [9]. Supporting proteome analyses confirmed that organic PM extracts induce a hierarchical oxidative stress response in macrophages and epithelial cells, in which the induction of electrophile-response element (EpRE) regulated genes (for example, heme oxygenase 1, catalase, and superoxide dismutase) at lower levels of oxidative stress prevented the more damaging pro-inflammatory and pro-apoptotic effects seen at higher levels of oxidative stress [10]. It is now widely recognized that oxidant injury is one of the principal mechanisms of PM-induced pulmonary inflammation and that this mechanism could also be applicable to the atherogenic effects of PM [11].

Atherosclerosis is a chronic vascular inflammatory process where lipid deposition and oxidation in the artery wall constitute a hallmark of the disease [12-17]. Infiltrating lipids come from low-density lipoprotein (LDL) particles that travel into the arterial wall and get trapped in a three-dimensional cagework of extracellular fibers and fibrils in the subendothelial space [18,19], where they are subject to oxidative modifications [20-22] leading to the generation of 'minimally modified' LDL (mm-LDL). Such oxidized LDL is capable of activating the overlying endothelial cells to produce pro-inflammatory molecules such as adhesion molecules, macrophage colony-stimulating factor (M-CSF) and monocyte chemotactic protein-1 (MCP-1) [23-25] that contribute to atherogenesis by recruiting additional monocytes and inducing macrophage differentiation [12,13,17]. We propose that PM-induced oxidative stress synergizes with oxidized lipid components to enhance vascular inflammation, leading to an increase in atherosclerotic lesions. Indeed, further LDL oxidation by ROS and lipoxygenases, myeloperoxidase, and secretory phospholipase can result in 'highly oxidized' LDL (ox-LDL) [17], taken up by macrophage scavenger receptors (for example, SR-A and CD36) to form foam cells [26]. Not only are mm-LDL and ox-LDL key components in the vicious cycle of oxidative stress and inflammation in the vascular wall [17,27], but we have shown that phospholipid oxidation products such as 1-palmitoyl-2-arachidonyl-*sn*-glycero-3-phosphorylcholine (ox-PAPC) lead to the upregulation of relevant gene clusters in human aortic endothelial cells [28]. In the lung, DEP chemicals may similarly lead to the regulation of gene groups in the vasculature that overlap or synergize with genes regulated by ox-PAPC.

We have found that exposure to PM in the ultrafine size range (particles smaller than 0.18 μm in aerodynamic diameter) resulted in increased systemic oxidative stress and greater atherosclerotic lesions in *apoE *null mice (unpublished work). These systemic vascular effects may be the result of synergy between oxidized phospholipids generated in circulating LDL particles and pollutant chemical that can be translocated or systemically absorbed from atmospheric nanoparticles [29,30]. We have explored this possible synergy between PM-bound chemicals and oxidized lipids by studying gene expression in human microvascular endothelial cells (HMEC). HMEC were treated with a pro-oxidative organic extract prepared from diesel exhaust particles (DEP), ox-PAPC or a combination of both. To assess the gene-expression profiles, we used Illumina microarrays. Apart from measuring differential expression between the treatment groups, we also clustered the genes into modules using weighted gene coexpression network analysis. We found that DEP extracts and ox-PAPC affected the expression of a large number of genes, and demonstrated synergistic effects on genes that play a role in antioxidant, inflammatory and unfolded protein response (UPR) pathways. We also examined the synergistic effect of ambient PM and oxidized lipids in *apoE *null mice fed a high-fat diet, demonstrating that similar pathways were activated *in vivo*.

## Results

### DEP and ox-PAPC upregulate HO-1 expression synergistically

**Figure 1:**
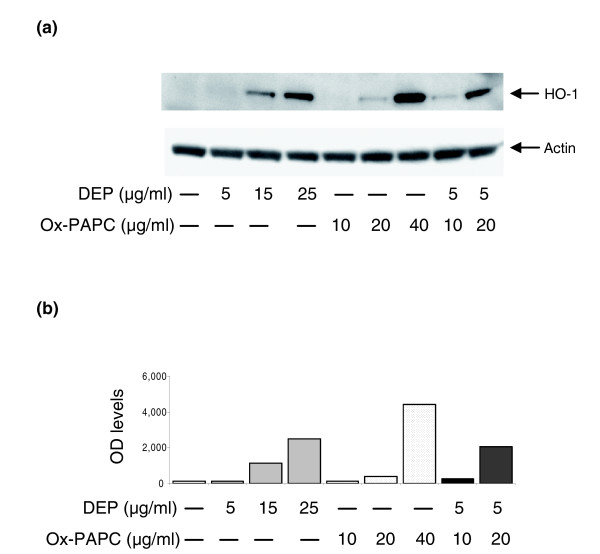
DEP and ox-PAPC induce HO-1 expression in HMEC. **(a) **Western blot. HMEC were treated with DEP, ox-PAPC or a combination of both at various concentrations. Mouse monoclonal anti-HO-1 and anti-β-actin antibodies were used to detect the relevant proteins as described in Materials and methods. **(b) **Densitometric analysis. The expression level of HO-1 protein in optical density (OD) units is shown. Similar levels of β-actin are shown in (a). Results are typical of one representative experiment (*n *= 4).

We have shown that treatment of human aortic endothelial cells (HAEC) with ox-PAPC leads to the generation of reactive oxygen species (ROS) and the activation of several molecular pathways, including EpRE regulated genes [28]. Diesel exhaust particles (DEP) have also been shown to elicit ROS production in pulmonary artery endothelial cells [31] and rat heart microvessel endothelial cells [32]. Because heme oxygenase-1 (HO-1) is an important oxidative stress sensor that is upregulated by both ox-PAPC [28,33,34] and DEP in endothelial cells [32], we investigated whether there was any additive or synergistic co-regulation in human microvascular endothelial cells (HMEC). We treated HMEC with ox-PAPC at concentrations of 10, 20, and 40 μg/ml; DEP at concentrations of 5, 15, and 25 μg/ml or DEP (5 μg/ml) plus ox-PAPC at concentrations of 10 or 20 μg/ml for 4 hours. Western blot analysis showed that induction of HO-1 expression by DEP and/or ox-PAPC was dose dependent (Figure 1a). Furthermore, HO-1 was synergistically co-regulated, as the co-treatment with both stimuli resulted in an expression level that was clearly greater than each stimulus alone or the sum of their response levels. Indeed, at a DEP dose of 5 μg/ml, the addition of ox-PAPC 20 μg/ml induced a HO-1 protein band density that was, respectively, 15-fold and 5-fold greater than the protein band densities corresponding to either DEP or ox-PAPC alone (Figure 1b).

### DEP and ox-PAPC regulate a large number of genes

**Figure 2:**
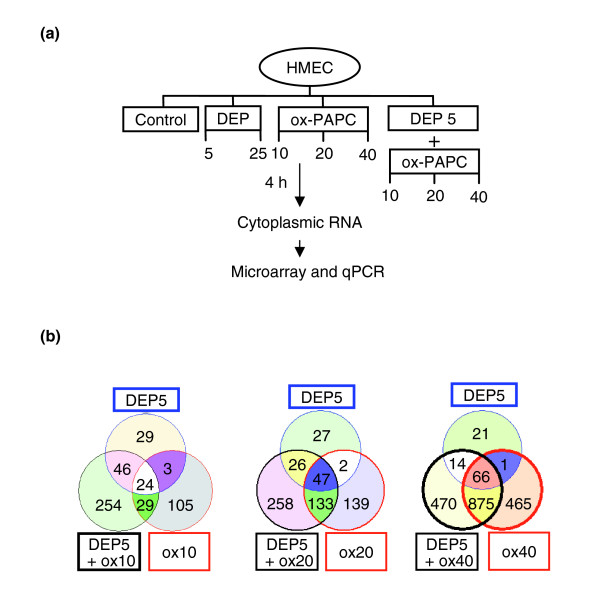
DEP and ox-PAPC induce a large number of genes in HMEC. **(a) **Experimental protocol. HMEC were treated in triplicate wells with DEP, ox-PAPC, or DEP + ox-PAPC at the various concentrations shown. Cells were harvested at 4 h and cytoplasmic RNA prepared. Illumina microarrays were run and the data confirmed by qPCR analysis of selected genes. **(b) **Venn diagrams of upregulated genes. The numbers of genes that were significantly upregulated (> 1.5-fold, *p *< 0.05) over controls (no treatment) by the various treatment are shown. The left Venn diagram summarizes the number of genes induced by DEP 5 μg/ml (DEP5), ox-PAPC 10 μg/ml (ox10) and DEP5 + ox10. The middle Venn diagram shows the number of genes induced by DEP5, ox-PAPC 20 μg/ml (ox20) and DEP5 + ox20. The right Venn diagram summarizes the number of genes induced by DEP5, ox-PAPC 40 μg/ml (ox40) and DEP5 + ox40. The total number of genes induced by a particular condition can be found by adding all values displayed within the circle corresponding to that condition. Values displayed in the circle intersections indicate the number of genes induced in common by the intersecting conditions.

We evaluated the transcriptomes of DEP- and ox-PAPC-regulated genes in HMEC and assessed their gene-expression profiles using Illumina microarray technology. The microarray data discussed in this publication have been deposited in the Gene Expression Omnibus [35] and are accessible through GEO Series accession number GSE6584. HMEC were treated in triplicate wells with DEP at concentrations of 5 and 25 μg/ml, ox-PAPC at concentrations of 10, 20, and 40 μg/ml or DEP at 5 μg/ml plus ox-PAPC at concentrations of 10, 20, and 40 μg/ml for 4 hours (Figure 2a). Illumina microarray analyses showed that ox-PAPC regulated a large number of genes in a dose-dependent fashion that was evident for both upregulated (Figure 2b) and downregulated genes (Additional data file 1), consistent with our previous reports [28]. Similarly, DEP treatment resulted in a significant and dose-dependent upregulation or downregulation of a number of genes. Thus, 25 μg/ml of DEP extract changed the expression profile of a significantly greater number of genes than DEP at 5 μg/ml (data not shown). More importantly, the combined treatment of 5 μg/ml DEP with various doses of ox-PAPC resulted in the altered expression of a greater number of genes than each corresponding dose of ox-PAPC alone (Figure 2b, and Additional data file 1). Altogether, 1,555 genes were significantly upregulated (> 1.5-fold, *p *< 0.05) by the three DEP and ox-PAPC treatment combinations. Notably, some genes were uniquely regulated by ox-PAPC and not by DEP; vice versa, some genes were regulated by DEP but not by ox-PAPC (Figure 2b).

### Synergistically regulated gene modules

**Figure 3:**
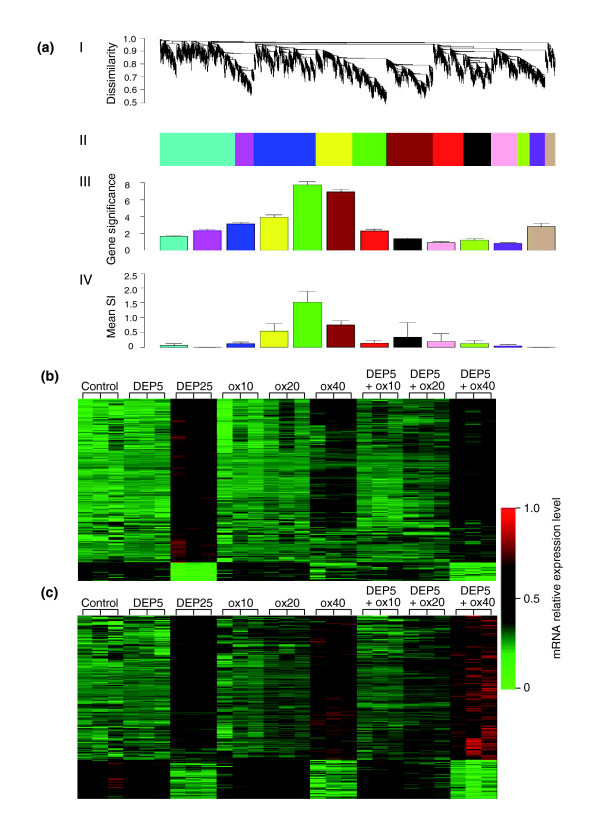
Gene coexpression network analysis. **(a) **The gene coexpression network. The 3,600 most varying genes were selected to construct a weighted gene coexpression network. I, The average linkage hierarchical clustering tree; II, clustered gene modules represented by different colors; III, gene significance of the individual modules. The green, brown and yellow modules were enriched in significant genes most highly correlated with the treatment conditions (*p *< 0.0001). Gene significance = -log (*p *value). IV, The synergistic gene enrichment. The mean synergistic indices (SI) of network genes that were upregulated by DEP, ox-PAPC and the corresponding combinatorial treatment of DEP plus ox-PAPC were calculated for each network module. The green, brown and yellow modules were also enriched in genes synergistically coregulated. Mean SI, mean synergistic index as defined in Materials and methods. **(b) **Heat map of the green module; **(c) **Heat map of the brown module. Expression levels of (b) 307 genes and (c) 426 genes are represented in the rows by color coding (green = low expression, red = high expression), in triplicate samples for each treatment condition (columns). Both modules show a clear synergistic/additive pattern where the combinatory treatments exhibited as a whole either a greater level of upregulation (towards red) in 274 genes (b) and 335 genes (c) at the top or downregulation (towards green) in 33 genes (b) and 91 genes (c) at the bottom, compared with the corresponding concentrations of DEP and ox-PAPC alone. Color scale is shown at the right of both heat maps, ranging from 0 (indicated by the green color at the bottom) to 1.0 (indicated by the red color at the top) as a reflection of the level of mRNA expression. DEP5 and DEP25, DEP 5 and 25 μg/ml, respectively; ox10, ox20, ox40: ox-PAPC 10, 20 and 40 μg/ml, respectively; DEP5 + (ox10, ox20 ox40): DEP 5 μg/ml + ox-PAPC 10, 20 and 40 μg/ml respectively.

We used weighted gene coexpression network analysis (WGCNA) to identify modules of highly coexpressed genes [36]. For computational reasons, we restricted the network analysis to the 3,600 genes that varied the most. As detailed in Materials and methods, we used unsupervised hierchical clustering to identify 12 modules of densely interconnected genes (Figure 3a, panels I, II) that were given unique color codes. Module-enrichment analysis showed that three modules (brown, green, and yellow) were significantly (*p *< 0.0001) enriched in genes regulated by the treatments (Figure 3a, panel III). In particular, the brown and the green modules were most highly enriched in genes that were differentially expressed by the treatments (Figure 3a, panel III). From the heat maps reflecting green and brown module gene expressions (Figure 3b,c), one can see that these genes are synergistically regulated by DEP and ox-PAPC. Remarkably, the yellow module also showed similar synergistic/additive gene response (Additional data file 2).

**Figure 4:**
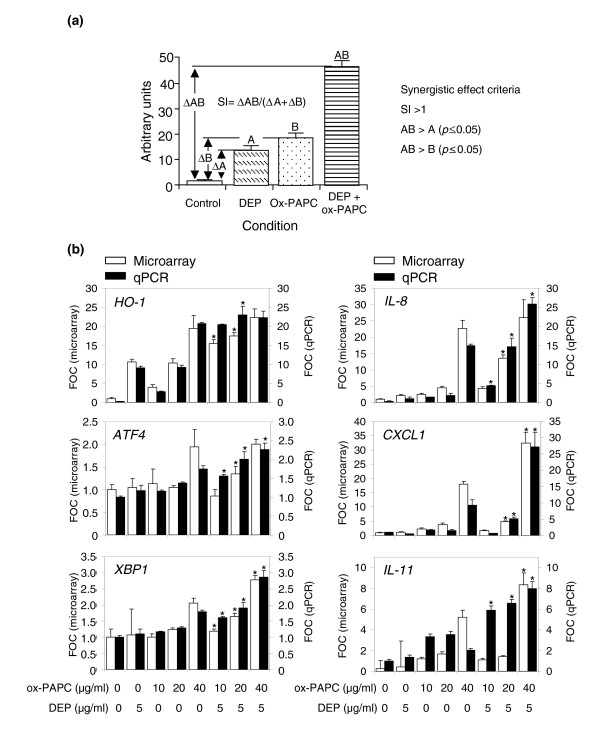
DEP and ox-PAPC co-regulate genes in a synergistic/additive fashion. **(a) **Synergistic index (SI). Synergy was defined as the presence of a co-regulatory effect by both DEP and ox-PAPC that was greater than the effects induced by either compound alone and greater than the sum of those individual effects. The following criteria for a synergistic effect were as follows: SI (ΔAB/(ΔA+ΔB) > 1; AB (mean) > A (mean), *p *≤ 0.05; AB (mean) > B (mean), *p *≤ 0.05, where ΔA is the difference in mean expression level between the DEP and the control samples, ΔB is the difference in mean expression level between the ox-PAPC and the control samples, and ΔAB is the difference in mean expression level between the DEP + ox-PAPC and the control samples. **(b) **mRNA expression levels of representative genes. Each graph displays the relative mRNA expression levels normalized by β_2_-microglobulin mRNA levels and expressed as fold control (FOC) for microarray (white bars, left-hand *y*-axis) and qPCR (black bars, right-hand *y*-axis) assessment of representative genes (*HO-1*, *IL-8*, *ATF4*, *CXCL1*, *XBP1*, *IL-11*). For ease of comparison, the qPCR scale was divided by factors of 3.5 (*HO-1*) and 3 (*IL-8*), respectively. In similar fashion, the microarray scale was divided by a factor of 4 (*IL-11*) to make the comparison easier. The asterisk indicates combinations of DEP + ox-PAPC that exhibited synergistic effects. The high consistence of microarray and qPCR analysis, conducted on triplicate samples from independent experiments, implies both technical and biological validation. Statistical analysis was performed by one-way ANOVA, Fisher PLSD.

To differentiate synergistically enhanced from additive gene responses during co-treatment with DEP and ox-PAPC, synergy was defined as follows. First, mean gene-expression levels were determined for the combination of DEP and ox-PAPC (mean AB); DEP only (mean A); ox-PAPC only (mean B); and the mean expression in controls (mean C). Second, we adjusted the mean expression levels in the treatment groups by subtracting the basal level as reflected in the control group: that is, we defined ΔAB = mean AB minus mean C, ΔA = mean A minus mean C, and ΔB = mean B minus mean C (Figure 4a). Third, we defined the synergistic index (SI) as follows, SI = ΔAB/(ΔA + ΔB). Because we were interested in positive synergistic effects, we considered a gene as synergistically expressed if the following criteria were met in at least one combinatorial treatment: SI > 1; AB (mean) > A (mean) (*p *= 0.05); and AB (mean) > B (mean) (*p *= 0.05) (Figure 4a). According to these criteria, 664 out of the 1,555 genes that were significantly upregulated (> 1.5 fold, *p *< 0.05) in the three DEP and ox-PAPC combinatorial conditions exhibited a synergistic effect. Of those 664 genes, 382 were present in the 3,600 most varying genes used for the network analysis. More significantly, 83% of these synergistically expressed genes were concentrated in the brown, green and yellow modules. These three modules also exhibited the highest modular mean SI (Figure 3a, panel IV). Thus, unsupervised clustering found modules (pathways) of synergistically expressed genes.

### Functional enrichment analysis of gene modules detects pathways related to vascular inflammation

**Figure 5:**
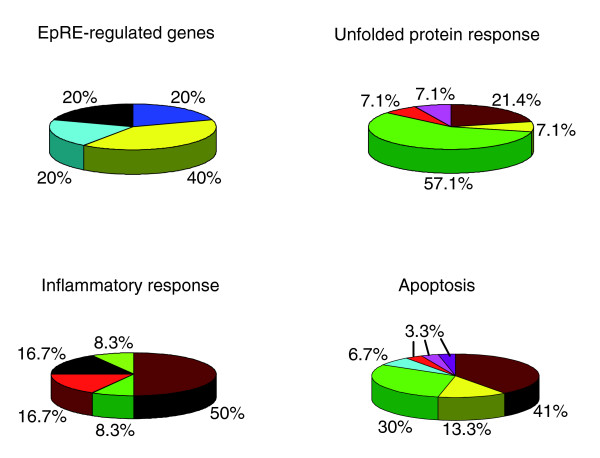
The distribution of genes for different pathways in the gene coexpression network modules. The 3,600 most varying genes were used for a weighted gene coexpression network construction and subjected to GO biological process pathway analysis using the EASE software [37]. Values shown are the percentage of pathway genes present in the coexpression network that are clustered in color-labeled network modules. The colors correspond to the color-labeled modules defined in Figure 3a.

To dissect the biological importance of genes upregulated synergistically by DEP and ox-PAPC, we studied the functional enrichment (using GO Ontology) of the 3,600 most varying genes, using the EASE software program [37]. Pathway analysis showed that the most varying genes were significantly enriched for EpRE, inflammatory response, UPR, immune response, cell adhesion, lipid metabolism, apoptosis, and protein folding genes (Additional data file 3). In particular, the three modules brown, green, yellow, comprising differentially expressed genes, were particularly enriched in these pathway genes (Figure 5, and Additional data files 4, 5). Indeed, these three modules concentrated around 40% of the EpRE genes, around 58% of the pro-inflammatory response genes, around 84% of the apoptosis pathway genes, and around 79% of the UPR genes that were present in the whole gene coexpression network (Figure 5, and Additional data files 4, 5). Interestingly, most of the pro-inflammatory response genes co-localized with activating transcription factor 4 (ATF4) in the brown module, a key mediator in the UPR signaling that we have previously reported as significantly induced by ox-PAPC in human aortic endothelial cells [28].

We validated our gene-expression data by quantitative PCR (qPCR) in the same set of samples analyzed by microarray analysis and in a set of samples from an independent experiment. Representative genes from various pathways were selected including EpRE-regulated genes (for example, *HO-1*, and selenoprotein S (*SELS*)), inflammatory response genes (for example, interleukin 8 (*IL-8*), and chemokine (C-X-C motif) ligand 1 (*CXCL1*)), immune-response genes (for example, interleukin 11 (*IL-11*)), UPR genes (for example, *ATF4*, heat-shock 70 kDa protein 8 (*HSPA8*), and X-box binding protein 1 (*XBP1*)), oxygen and ROS metabolism genes (for example, dual-specificity phosphatase 1 (*DUSP1*), and PDZ and LIM domain 1 (*PDLIM1*)). All of these genes were synergistically co-regulated by DEP and ox-PAPC in at least one combinatorial treatment (Figure 4b, and Additional data file 6). qPCR could confirm 91% of the synergistic effects that were revealed by microarray technology.

### DEP and ox-PAPC co-regulatory effects have *in vivo *correlates

**Figure 6:**
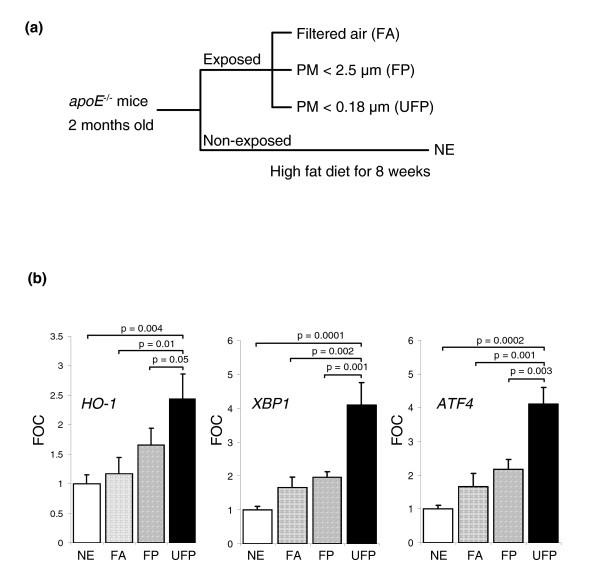
Ambient ultrafine PM chemicals enhance *in vivo *expression of genes related to vascular inflammation. **(a) **Experimental protocol. Two-month-old male *apoE *null mice fed a high-fat diet were exposed for a total of 120 h (5 h/day, 3 days/week for 8 weeks) to concentrated ultrafine particles (UFP), concentrated PM_2.5 _(FP), filtered air (FA) or not exposed (NE). **(b) **Hepatic gene expression levels. Gene expression was determined by qPCR of mRNA prepared from liver homogenates. UFP-exposed mice exhibited marked upregulation of *HO-1 *(left), *XBP1 *(center) and *ATF4 *(right). Values were normalized by β-actin mRNA levels and expressed as fold control (FOC). Five samples per group were assayed in duplicates. Statistical analysis was performed by one-way ANOVA, Fisher PLSD.

We investigated whether the DEP and ox-PAPC synergistic effects occurred *in vivo *by evaluating the expression of representative genes in liver tissue homogenates of apoE-null mice, fed a high fat diet (HFD) and exposed to PM in a mobile animal laboratory in downtown Los Angeles. Oxidized lipids play an important role in the generation of vascular injury in these hypercholesterolemic animals [38]. Mice were exposed to concentrated ultrafine particles (UFP = particles < 0.18 μm), which in an urban environment are mostly comprised of DEP, and compared to animals exposed to concentrated PM_2.5 _(particles with an aerodynamic diameter < 2.5 μm, also known as fine particles or FP) or filtered-air (FA), or compared to mice that were left unexposed. Because we have previously noted that PM induces systemic oxidative stress effects in these animals, most noticeably in the liver, hepatic tissue was assayed for mRNA expression of HO-1, as well as two key UPR transcription factors, XBP1 and ATF4. UFP-exposed animals exhibited a significant upregulation (*p *< 0.05) of all three genes in comparison with FP, FA, and unexposed mice (Figure 6). These results indicate that the synergistic effects predicted by our *in vitro *studies have important *in vivo *outcomes, in which pro-oxidative PM chemicals may gain access to the systemic circulation from the lung and may then be able to synergize with circulating ox-LDL.

## Discussion

We have used HMEC as a representative cell type to study the synergistic effects of DEP chemicals and ox-PAPC on inflammatory gene expression. We found that DEP and ox-PAPC could co-regulate a large number of genes that are involved in atherosclerosis and vascular injury associated with ambient PM exposures. This includes the upregulation (> 1.5-fold, *p *< 0.05) of 1,555 genes by a low dose of DEP combined with three different doses of ox-PAPC (Figure 2b). In addition, the same treatment resulted in downregulation of 759 genes (Additional data file 1). Remarkably, 43% of all upregulated genes exhibited a pattern of synergy in which the combination resulted in a bigger response than either of the individual stimuli. By using a module enrichment analysis [36] based on the 3,600 most varying genes, we identified three groups of genes (modules) that were most highly correlated to the treatments (*p *< 0.0001) and were especially enriched in synergistically expressed genes. Further analysis of these three modules demonstrated that the gene clusters belonged to pathways relevant to vascular inflammation, including atherosclerosis. Moreover, the synergistic upregulation of selected EpRE, pro-inflammatory, apoptotic and UPR genes could be confirmed by qPCR analysis. The *in vivo *relevance of this gene-clustering analysis was established by comparing gene expression in livers of hypercholesterolemic mice exposed to UFPs versus mice that were exposed to FPs or FA or were left unexposed. UFP-exposed animals indeed exhibited significantly increased expression of EpRE and UPR genes, predicted by the *in vitro *synergy between DEP and ox-PAPC in HMEC (Figure 6).

Cumulative evidence supports the association of ambient air pollution with daily total and cardiovascular mortality [39,40], an association best established for the level of ambient PM [41,42]. Both experimental animal [43,44] and human epidemiological work [45] have shown that exposure to ambient PM promotes atherosclerosis, a disease process in which the endothelial responses are of paramount importance. Notably, small particles appear to have a bigger impact on atherogenesis than larger particles [46]. Thus, our gene-expression data are of considerable importance in understanding how ambient air pollution might contribute to endothelial injury and to atherosclerosis. While there is still considerable uncertainty and debate about the mechanism(s) of cardiovascular injury by PM, it is becoming increasingly clear that PM exerts pro-oxidative and pro-inflammatory effects in the lung that can also spill over to the systemic circulation. The systemic effects could result either from the systemic release of inflammatory mediators from the lung or from the possible direct access of particles or chemicals to the systemic circulation. In either scenario, the interaction of PM components with the vascular endothelium in the lung or in the systemic circulation may be relevant in the generation of systemic vascular effects. We propose that such vascular effects are magnified by their interaction with oxidized phospholipids generated in LDLs or in the membranes of vascular endothelial cells. While it is not possible to reconcile the *in vitro *and *in vivo *dosimetry in the case of endothelial cells, we have previously reported in macrophages and bronchial epithelial cells that *in vitro *DEP extract concentrations in the dose range 1-100 mg/ml correspond to realistic particle concentrations at hotspots of deposition in the respiratory tract [47]. Thus, it is possible to achieve particle doses at microdomains that are equivalent to the particle dose range that can be achieved if the dose is recalculated from mass/volume to mass per unit surface area. It is possible that similar flow-directed hotspots could exist in the cardiovascular tree, for example the ostia of the coronary arteries.

UFPs are rich in organic chemicals such as polycyclic aromatic hydrocarbons (PAH) and quinones (Additional data files 7-9). These chemicals participate in the generation of ROS by their redox cycling as well as possibly through a perturbation of mitochondrial function [48]. We and others have shown that such PM-mediated oxidative stress can trigger cytoprotective antioxidant responses in bronchial epithelial cells, macrophages [49], pulmonary artery endothelial cells [31], and rat heart microvessel endothelial cells [32]. This response may represent the first level of a hierarchical oxidative stress response, as demonstrated in macrophages and epithelial cells [49]. Failure of the antioxidant response to maintain redox equilibrium could subsequently lead to pro-inflammatory and cytotoxic/apoptotic effects at high levels of oxidative stress [49]. Oxidized phospholipids such as ox-PAPC, generated in the LDL particles or cell membranes, also exert oxidative stress effects in human aortic endothelial cells [34]. Here we show that oxidative stress elicited by PM chemicals synergizes with the effect of ox-PAPC, possibly because they target different intracellular activation pathways.

Endothelial cell responses to oxidative stress are of fundamental importance in atherogenesis. It is possible that endothelial cells also exhibit a similar hierarchical response as described in macrophages and epithelial cells in response to pro-oxidative DEP chemicals [49]. ROS generation may lead to a decreased intracellular concentration of reduced glutathione (GSH) and thus to a decreased ratio of GSH to GSSG (oxidized glutathione) that can act as a sensor and trigger additional cellular responses. One example is the initiation of a protective cellular response by the transcription of EpRE-regulated genes [50]. Indeed, we have shown that DEP and ox-PAPC synergize in the induction of genes such as HO-1 (Figure 4b), SELS, NADPH quinone oxidoreductase-1 (NQO-1) and superoxide dismutase 1 (SOD1) (Additional data file 6). EpRE-regulated gene expression is also evident *in vivo*, as livers from UFP-exposed *apoE *null mice exhibited significantly increased HO-1 levels in comparison with animals exposed to FA or left unexposed (Figure 6). Interestingly, UFP was able to trigger HO-1 expression despite the overwhelming stimulus resulting from a high-fat diet in ApoE-deficient animals.

According to the hierarchical oxidative stress paradigm, higher levels of oxidative stress may overwhelm the cytoprotective and antioxidant effects of the first tier of response. This could lead to the initiation of injurious cellular effects as a result of the activation of pro-inflammatory mitogen-activated protein kinase (MAPK) and NF-κB signaling cascades [49]. In accordance with this concept, we show that both DEP and ox-PAPC could induce the synergistic expression of IL-8, CXCL1 and IL-11 mRNA (Figure 4b, and Additional data file 6), all of which are relevant to vascular inflammation [51]. Such synergistic regulation is more evident at the higher doses of ox-PAPC, which supports the hierarchical oxidative stress model. One possible explanation for this synergy is that DEP and ambient PM induce MAPK and NF-κB activation [52], whereas ox-PAPC may act through the separate, but related, UPR pathway in endothelial cells [28]. It is interesting therefore, that UPR genes such as *XBP1*, *ATF3 *and *ATF4 *could be seen to be synergistically upregulated by DEP plus ox-PAPC (Figure 4b, and Additional data file 6). We and others have previously shown that ox-PAPC upregulates UPR genes such as *ATF3 *and *ATF4 *in HAECs with concurrent expression in atherosclerotic lesions [28,53]. In addition, ambient UFPs upregulate *ATF4 *and *XBP1 *expression *in vivo *(Figure 6b), suggesting that the UPR pathway may play a role in the promotion of vascular injury by PM.

An important step in understanding how ambient PM promotes endothelial cell dysfunction and atherosclerosis is to dissect the mechanisms of how DEP and ox-PAPC synergize in the induction of relevant genes. Such synergy may be accomplished by various mechanisms, such as recognition of different receptors, targeting of different intracellular signaling cascades, and activity on different promoter elements of synergistic genes. The identification of such mechanisms will help clarify the means by which ambient PM result in vascular dysfunction.

## Materials and methods

### Cell cultures

A human microvascular endothelial cell (HMEC) line, originally isolated from six human foreskins, was obtained from Francisco Candal (Centers for Disease Control and Prevention, Atlanta, GA) and cultured as described previously [54]. Cells were treated in triplicate wells with DEP (5 or 25 μg/ml), ox-PAPC (10, 20 or 40 μg/ml), or DEP 5 μg/ml + ox-PAPC (10, 20, or 40 μg/ml) in media containing 1% FBS (Irvine Scientific, Santa Ana, CA). ox-PAPC was generously provided by Judith Berliner (University of California Los Angeles, CA), who has described a detailed mass spectrometric analysis of the material [55,56]. ox-PAPC consists of a mixture of oxidized phospholipids that include as main components 1-palmitoyl-2-(5-oxovaleroyl)-*sn*-glycero-3-phosphorylcholine (POVPC), 1-palmitoyl-2-glutaroyl-*sn*-glycero-3-phosphorylcholine (PGPC), and 1-palmitoyl-2-(5,6)-epoxyisoprostane E_2_-*sn*-glycero-3-phosphocholine (PEIPC). Diesel exhaust particles were a gift from Masaru Sagai (National Institute for Environmental Studies, Tsukuba, Japan). These particles were collected from the exhaust in a 4JB1-type LD, 2.74 l, 4-cylinder Isuzu diesel engine under a load of 10 torque onto a cyclone impactor equipped with a dilution tunnel constant volume sampler [57,58]. DEP was collected on high-capacity glass-fiber filters, from which the scraped particles were stored as a powder in a glass container under nitrogen gas. The particles consist of aggregates in which individual particles are less than 1 μm in diameter. The chemical composition of these particles, including PAH and quinone analysis, as well assessment of their oxidant potential by the dithiothreitol (DTT) assay was previously described [9,57-59]. DEP methanol extracts were prepared as previously described [9,57,59]. Briefly, 100 mg DEP were suspended in 25 ml methanol and sonicated for 2 min. The DEP methanol suspension was centrifuged at 2,000 rpm for 10 min at 4°C. The methanol supernatant was transferred to a pre-weighed polypropylene tube and dried under nitrogen gas. The tube was re-weighed to determine the amount of methanol extractable DEP components. Dried DEP extract was then dissolved in DMSO at a concentration of 100 μg/μl. The aliquots were stored at -80°C in the dark until used. DEP components are shown in Additional data files 7-9. The chemical composition of this extract, including the presence of the redox cycling organic substances such as polycyclic aromatic hydrocarbons and quinones, has been previously described by us [58].

### Western blot analysis

HMEC were harvested and lysed in lysis buffer (25 mM Hepes pH 7.4, 50 mM β-glycerophosphate, 1 mM para-nitrophenolphosphate, 2.5 mM MgCl_2_, 1% Triton, complete Protease Inhibitor Cocktail Tablets (Roche Applied Science, Indianapolis, IN)). Protein samples (25 μg/well) in SDS loading buffer were subjected to 4-12% SDS-polyacrylamide gel electrophoresis (PAGE) and transferred to nitrocellulose membrane (Bio-Rad, Hercules, CA). The membrane was blocked with 5% dry milk and 0.1% Tween 20 (USB, Cleveland, OH). Mouse monoclonal anti-HO-1 antibody (StressGen Biotech, Victoria, Canada) and mouse monoclonal anti-β-actin antibody (Abcam, Cambridge, MA) were used as primary antibodies at 1:1,000 dilution overnight, respectively. Anti-mouse IgG horseradish peroxidase-linked secondary antibody (Amersham Biosciences, Piscataway, NJ) was used as secondary antibody at 1:2,000 dilution for 1 h. Chemiluminescent signals were detected by enhanced chemiluminescence assay (Pierce, Rockford, IL). Protein expression levels were determined using a densitometer (Kodak Digital Science 1D Analysis Software; Kodak, Rochester, NY).

### RNA preparation and expression microarray analyses

HMEC were cultured, treated in triplicate wells and harvested as described. Cytoplasmic RNA was isolated by RNeasy kit (Qiagen, Valencia, CA) and analyzed on an Agilent 2100 Bioanalyzer (Agilent, Palo Alto, CA) to assess RNA integrity. Biotin-labeled cRNA was synthesized by the Total prep RNA amplification kit from Ambion (Austin, TX). cRNA was quantified and normalized to 77 ng/μl, and then 850 ng was hybridized to Beadchips (Beadchip 8X1, Illumina, San Diego, CA) that contain probes for around 23,000 transcripts. The hybridized Beadchips were scanned by an Illumina BeadScan confocal scanner and analyzed by Illumina's BeadStudio software, version 1.5.1.3. cRNA synthesis, hybridization and scanning were performed at the UCLA Illumina microarray core facility. The microarray data was normalized by the rank invariant method and analyzed using BeadStudio software.

### Quantitative real-time PCR

Cytoplasmic RNA was isolated from cells using RNeasy (Qiagen). One microgram of total RNA was reverse transcribed using random hexamer primers and Superscript-III reverse transcriptase (Invitrogen, Carlsbad, CA). Quantitative RT-PCR (qPCR) was performed using iQ and SYBR Green detection kits (Bio-Rad, Hercules, CA). Primers were designed by PrimerQuest software (Integrated DNA Technololgies, Coralville, IA). PCR conditions were three 3-min steps of 94°C and 40 cycles of 94°C for 15 sec, 60°C for 30 sec, and 72°C for 30 sec. Expression levels were determined from cycle thresholds using a standard curve, normalized to human β_2_-microglobulin or mouse β-actin expression levels and expressed as fold-control.

### Weighted gene coexpression network construction

We followed the method for constructing a weighted gene coexpression network previously reported by us [36]. Briefly, the absolute value of the Pearson correlation coefficient was calculated for all pairwise comparisons of gene-expression values across all microarray samples. The Pearson correlation matrix was then transformed into an adjacency matrix *A *- that is, a matrix of connection strengths using a power function. Thus, the connection strength *a*_*ij *_between gene expressions *x*_*i *_and *x*_*j *_and was defined by *a*_*ij *_= |*cor*(*x*_*i*_, *x*_*j*_)|^*β*^. The network connectivity (k_all_) of the *i*th gene is the sum of the connection strengths with the other genes, that is, $k_{i}=\sum_{u\neq i}a_{iu}$. This summation performed over all genes in a particular module is the intramodular connectivity (k_in_). We chose a power of *β *= 6 based on the scale-free topology criterion [36] but our findings are highly robust with respect to this choice.

### Network module identification

Modules are defined as sets of genes with high 'topological overlap' [36,60]. The topological overlap measure can serve as an important filter to counter the effects of spurious or missing connections between network nodes. Specifically the topological overlap between genes *i *and *j *is written as $\omega_{ij}=\frac{l_{ij}+a_{ij}}{\min\{k_{i},k_{j}\}+1-a_{ij}}$

where, $l_{ij}=\sum_{u\neq i,j}a_{iu}a_{uj}$denotes the number of nodes to which both *i *and *j *are connected, and *u *indexes the nodes of the network. Because hierarchical clustering takes a dissimilarity measure as input, we defined a topological overlap-based dissimilarity measure as follows $d_{ij}^{\omega }=1-\omega_{ij}$. We defined modules as the branches of the resulting hierarchical clustering tree. We used average linkage hierarchical clustering as implemented in the R software [61].

### Module enrichment analysis

On the basis of the treatments with DEP, ox-PAPC, or DEP plus ox-PAPC, gene significance (GS) of the *i*th gene-expression profile *x*_*i *_was defined as

GS(*i*) = -log_10_(*p *value(*i*))

where the *p *value was computed using analysis of variance (F-statistic). An important step in gene network analysis is to study the biological relevance of network modules. To assess whether the modules were related to the treatments, we defined a module significance measure on the basis of gene significance measure. Specifically, we define a measure of module significance by the mean gene significance in the *q*th module, that is

$$ModuleEnrichment_{q}=\frac{\sum_{i=1}^{n_{q}}GS_{i}}{n_{q}}$$

where *i *indexes the genes in the *q*th module and *n*_*q *_denotes the module size. By considering the module significance measure in our applications, we observed that certain modules (green and brown modules) were enriched with differentially expressed genes. Similarly, the synergistic index (see below) gives rise to a module synergy measure.

### Assessment of synergy

Synergy was defined as the presence of a co-regulatory effect by both DEP and ox-PAPC that was greater than the effects induced by either compound alone and greater than the sum of those individual effects. To differentiate synergistically enhanced from additive gene response in those cases where there was upregulation over the untreated samples (controls), we developed a synergistic index (SI) defined as SI = ΔAB/(ΔA+ΔB) where ΔAB was the difference in between gene mean expression levels shown by a DEP plus ox-PAPC combinatorial treatment and controls, ΔA was the differential expression in between mean DEP and mean controls and ΔB was the differential expression in between the corresponding concentration of mean ox-PAPC and mean controls (Figure 4a). Such a SI was used for both microarray intensity and qPCR readouts. Synergy in upregulated genes required the concurrence of three criteria: SI > 1; AB (mean) > A (mean) (*p *= 0.05); and AB (mean) > B (mean) (*p *= 0.05). The enrichment of modules in synergistic genes was assessed by the determination of a modular mean SI, where non-synergistic genes were assigned a value of zero.

### Exposure of *apoE *null mice to ambient PM

Two-month-old male C57BL/6J *apoE *null mice (Jackson Laboratory, Bar Harbor, ME) were placed on a high-fat diet and exposed to concentrated ambient particles (CAP) for a total of 120 hours over a 56-day period (5 hours per day, 3 days per week for 8 weeks). Animals were euthanized 24 h after completion of the last CAPs exposure, and aortic roots and livers were harvested. Animals in the unexposed (NE) group were kept in the UCLA vivarium, and the mice destined for CAP exposure were transported to the mobile animal laboratory in downtown Los Angeles, close (approximately 300 m) to the 110 freeway. This mobile research laboratory (AirCARE1) is equipped with state-of-the-art research capabilities as previously described [62]. Mice were housed in filter-top cages under temperature- and humidity-controlled conditions. Exposures took place in custom-designed exposure chambers [63,64] that were connected to a particle-concentration-enrichment system or to a source of purified, filtered air (FA) [65]. Other than the NE group, there were three groups (17-18 mice per group) that were exposed to FA, CAP less than 2.5 μm in aerodynamic diameter (FP) and CAP less than 0.18 μm in aerodynamic diameter (UFP). Particle mass concentration and elemental CAP composition were determined as previously described [65]. Average FP mass, FP particle number concentration, and FP particle enrichment factor were 361.95 μg/m^3^, 2.7 × 10^5 ^particles/cm^3^, and 13.8-fold, respectively. Average UFP mass, UFP particle number concentration, and UFP particle enrichment factor were 128.55 μg/m^3^, 3.24 × 10^5 ^particles/cm^3^, and 16.5-fold, respectively. Experimental protocols were approved by the animal research committee at UCLA.

### Statistical analysis

For the microarray gene-expression analysis, the two-tail Student *t*-test embedded within the BeadStudio software was used. For the assessment of synergy, we used the F-test for multiple comparisons and values were considered significant at a *p *< 0.05. For qPCR analysis, we used ANOVA with two-tail Fisher PLSD post-hoc analysis. Differences were considered statistically significant at *p *< 0.05.

## Additional data files

The following additional data files are available online with this paper. Additional data file 1 shows the number of genes that DEP and ox-PAPC significantly downregulate. Additional data file 2 shows the heat map of the yellow module, where a pattern of synergistic/additive interaction is noted. Additional data file 3 contains selected pathway analysis on the total number of genes that were significantly regulated by DEP and/or ox-PAPC. Additional data file 4 shows the distribution of genes for particular pathways in the gene coexpression network modules. Additional data file 5 contains the list of genes that exhibited a synergistic mode of regulation in the gene network. Additional data file 6 shows SIs of representative genes as determined from microarray and qPCR data. Additional data file 7 contains the recovery of major organic fractions from 1 g DEP. Additional data file 8 contains the content of polycyclic aromatic hydrocarbons in crude DEP extract and fractions. Additional data file 9 contains the quinone content in crude DEP extract and fractions.

## Supplementary Material

Additional data file 1 The number of genes that DEP and ox-PAPC significantly downregulate. Click here for file

Additional data file 2 Heat map of the yellow module, where a pattern of synergistic/additive interaction is noted. Click here for file

Additional data file 3 Selected pathway analysis on the total number of genes that were significantly regulated by DEP and/or ox-PAPC. Click here for file

Additional data file 4 The distribution of genes for particular pathways in the gene coexpression network modules. Click here for file

Additional data file 5 A list of genes that exhibited a synergistic mode of regulation in the gene network. Click here for file

Additional data file 6 SIs of representative genes as determined from microarray and qPCR data. Click here for file

Additional data file 7 The recovery of major organic fractions from 1 g DEP. Click here for file

Additional data file 8 The content of polycyclic aromatic hydrocarbons in crude DEP extract and fractions. Click here for file

Additional data file 9 The quinone content in crude DEP extract and fractions. Click here for file
