# Supplementary material for: Air-pollutant chemicals and oxidized lipids exhibit genome-wide synergistic effects on endothelial cells
Source: Genome Biol. 2007 Jul 26;8(7):R149. doi: 10.1186/gb-2007-8-7-r149 (PMC2323217; doi:10.1186/gb-2007-8-7-r149)
Supplement: Additional data file 1 — The number of genes that DEP and ox-PAPC significantly downregulate. [file gb-2007-8-7-r149-S1.pdf]

Additional data file 1. DEP and ox-PAPC downregulate a significant number of genes

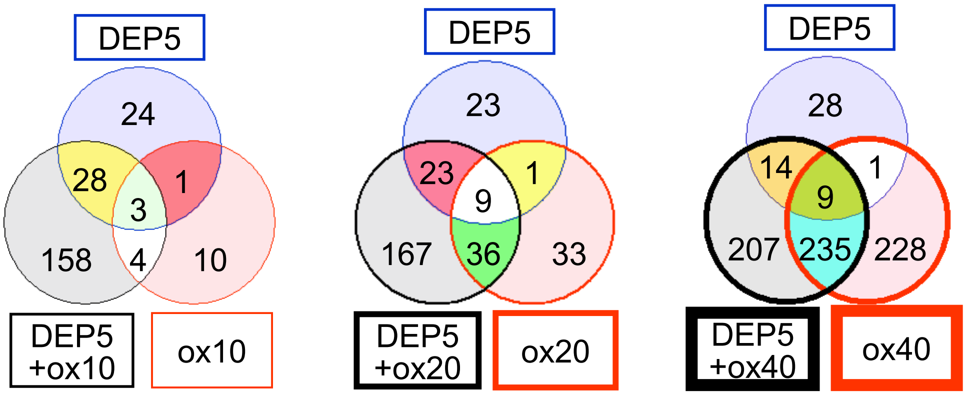

The number of genes (n) that were significantly downregulated (>1.5 fold, $p<0.05$) by DEP at 5 µg/ml, ox-PAPC (10, 20 or 40 µg/ml) or DEP 5 µg/ml + ox-PAPC (10, 20 or 40 µg/ml) over controls (no treatment) are represented in Venn diagrams. The total number of genes inhibited by a condition can be found by adding all values displayed within the circle corresponding to that condition. Values displayed in the circle intersections indicate the number of genes inhibited by the intersecting conditions in common. Left Venn diagram summarizes the number of genes inhibited by DEP 5 µg/ml, ox-PAPC 10 µg/ml and DEP 5 µg/ml + ox-PAPC 10 µg/ml. Middle Venn diagram shows the number of genes inhibited by DEP 5 µg/ml, ox-PAPC 20 µg/ml and DEP 5 µg/ml + ox-PAPC 20 µg/ml. Right Venn diagram summarizes the number of genes inhibited by DEP 5 µg/ml, ox-PAPC 10 µg/ml and DEP 5 µg/ml + ox-PAPC 40 µg/ml. DEP5: DEP 5 µg/ml; ox10, ox20 and ox40: ox-PAPC 10, 20 and 40 µg/ml respectively; DEP + (ox10, ox20 and ox40): DEP 5 µg/ml + ox-PAPC 10, 20 and 40 µg/ml respectively.
