# Supplementary material for: Air-pollutant chemicals and oxidized lipids exhibit genome-wide synergistic effects on endothelial cells
Source: Genome Biol. 2007 Jul 26;8(7):R149. doi: 10.1186/gb-2007-8-7-r149 (PMC2323217; doi:10.1186/gb-2007-8-7-r149)
Supplement: Additional data file 2 — Heat map of the yellow module, where a pattern of synergistic/additive interaction is noted. [file gb-2007-8-7-r149-S2.pdf]

**Additional data file 2.** Yellow module exhibits a synergistic/additive pattern

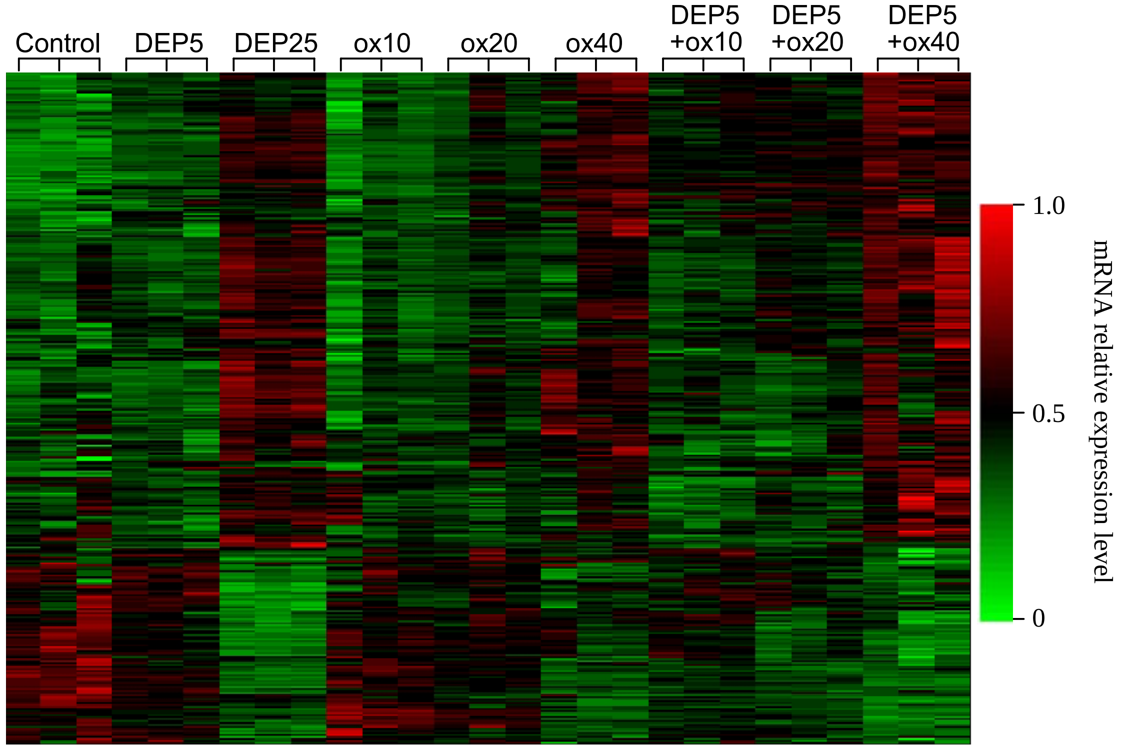


Expression level of the 337 genes (rows) is represented by color coding (green= low, red= high) in triplicate samples for each treatment condition (columns). Module shows a clear synergistic/additive pattern where the combinatory treatments exhibited either a greater level of upregulation (towards red) in 238 genes at the top or downregulation (towards green) in 99 genes at the bottom, in comparison with the corresponding concentrations of DEP and ox-PAPC alone. Color scale is shown at the right of the heat map, ranging from 0 (very bottom green) as the lowest to 1.0 (very top red) as the greatest relative gene mRNA expression level. DEP5 and DEP25: DEP 5 and 25 µg/ml respectively; ox10, ox20, ox40: ox-PAPC 10, 20 and 40 µg/ml respectively; DEP5 + (ox10, ox20 ox40): DEP 5 µg/ml + ox-PAPC 10, 20 and 40 µg/ml respectively.
