# Supplementary material for: Air-pollutant chemicals and oxidized lipids exhibit genome-wide synergistic effects on endothelial cells
Source: Genome Biol. 2007 Jul 26;8(7):R149. doi: 10.1186/gb-2007-8-7-r149 (PMC2323217; doi:10.1186/gb-2007-8-7-r149)
Supplement: Additional data file 3 — Selected pathway analysis on the total number of genes that were significantly regulated by DEP and/or ox-PAPC. [file gb-2007-8-7-r149-S3.doc]

**Additional data file 3**. Gene pathway analysis

| Pathways | DEP (µg/ml) | | ox-PAPC (µg/ml) | | | DEP 5 µg/ml + | | |
| --- | --- | --- | --- | --- | --- | --- | --- | --- |
|  |  |  |  |  |  | ox-PAPC (µg/ml) | | |
|  | 5 | 25 | 10 | 20 | 40 | 10 | 20 | 40 |
| All (total) | 158 | 1891 | 179 | 400 | 1974 | 546 | 693 | 1980 |
| Apoptosis | 4 | 60 | 5 | 18 | 67 | 24 | 31 | 70 |
| Cell adhesion | 3 | 27 | 10 | 14 | 42 | 20 | 20 | 50 |
| Inflammatory response | 1 | 21 | 4 | 5 | 19 | 6 | 7 | 18 |
| Lipid metabolism | 4 | 59 | 4 | 12 | 64 | 19 | 20 | 62 |
| Protein folding | 3 | 27 | 0 | 4 | 21 | 8 | 14 | 28 |
| Ubiquitin-dependent protein catabolism | 1 | 14 | 1 | 4 | 19 | 5 | 6 | 17 |
| Immune response | 5 | 52 | 7 | 17 | 54 | 20 | 26 | 59 |

Gene expression profile was analyzed by BeadStudio software (**Illumina, Inc.** San Diego, CA). The total number of genes that exhibited significant (p<0.05) up and/or downregulation (>1.5 fold) over controls are displayed in the first row. Such differentially expressed genes were subjected to pathway analysis by EASE software. The numbers of differentially expressed genes (>1.5 fold) belonging to each pathway are shown
