# Supplementary material for: Air-pollutant chemicals and oxidized lipids exhibit genome-wide synergistic effects on endothelial cells
Source: Genome Biol. 2007 Jul 26;8(7):R149. doi: 10.1186/gb-2007-8-7-r149 (PMC2323217; doi:10.1186/gb-2007-8-7-r149)
Supplement: Additional data file 4 — The distribution of genes for particular pathways in the gene coexpression network modules. [file gb-2007-8-7-r149-S4.doc]

**Additional data file 4**. Pathway genes distribution in the Gene co-expression network modules.

| Module | EpRE gene | Apoptosis | Cell adhesion | Inflammatory response | Lipid Metabolism | Protein folding | Unfolded Protein   Response | Ubiquitin-dependent   protein catabolism | Immune response |
| --- | --- | --- | --- | --- | --- | --- | --- | --- | --- |
| Blue | 1 |  | 4 |  | 2 | 1 |  | 4 |  |
| Brown |  | 12 | 7 | 6 | 1 | 1 | 3 |  | 11 |
| Yellow | 2 | 4 | 3 |  | 5 | 2 | 1 | 3 |  |
| Green |  | 9 | 6 | 1 | 4 | 17 | 8 | 1 | 7 |
| Turquoise | 1 | 2 | 1 |  |  |  |  |  | 1 |
| Red |  | 1 | 2 | 2 | 1 | 1 | 1 |  | 4 |
| Black | 1 |  |  | 2 | 1 | 1 |  |  | 3 |
| Pink |  | 1 |  |  |  |  | 1 | 1 | 1 |
| Magenta |  |  |  |  |  |  |  | 2 |  |
| Purple |  | 1 |  |  |  |  |  | 3 | 1 |
| Greenyellow |  |  | 1 | 1 |  | 1 |  | 1 | 1 |
| Tan |  |  |  |  |  |  |  |  | 2 |

3600 most varying genes were used for a weighted gene co-expression network construction and subjected to Go Biological Process Pathway analysis by the EASE software. Values shown are the number of pathway genes clustered in each color-labeled network module.
