# Supplementary material for: Air-pollutant chemicals and oxidized lipids exhibit genome-wide synergistic effects on endothelial cells
Source: Genome Biol. 2007 Jul 26;8(7):R149. doi: 10.1186/gb-2007-8-7-r149 (PMC2323217; doi:10.1186/gb-2007-8-7-r149)
Supplement: Additional data file 5 — A list of genes that exhibited a synergistic mode of regulation in the gene network. [file gb-2007-8-7-r149-S5.doc]

# Additional data file 5. Pathway genes synergistically co-regulated by DEP and ox-PAPC

| **GENE CATEGORIES** | **Module** | **GENE NAME** | **ACCESSION NUMBER** |
| --- | --- | --- | --- |
|
| **EpRE regulated genes** |  |  |  |
| CTSB | blue | cathepsin B | NM_001908.2 |
| SOD1 | black | superoxide dismutase 1 | NM_000454.3 |
| HMOX1 | yellow | heme oxygenase 1 | NM_002133.1 |
| NQO1 | turquoise | NAD(P)H dehydrogenase, quinone 1 | NM_000903.1 |
| SELS | yellow | selenoprotein S | NM_018445.4 |
|  |  |  |  |
| Apoptosis |  |  |  |
| PMAIP1 | brown | phorbol-12-myristate-13-acetate-induced protein 1 | NM_021127.1 |
| BAG3 | green | BCL2-associated athanogene 3 | NM_004281.2 |
| CASP4 | green | caspase 4 | NM_001225.2 |
| IER3 | brown | immediate early response 3 | NM_003897.2 |
| DAP3 | yellow | death associated protein 3 | NM_004632.2 |
| AXUD1 | brown | AXIN1 up-regulated 1 | NM_033027.2 |
| CDKN1A | brown | cyclin-dependent kinase inhibitor 1A | NM_000389.2 |
| FAF1 | yellow | Fas (TNFRSF6) associated factor 1 | NM_131917.1 |
| SH3GLB1 | turquoise | SH3-domain GRB2-like endophilin B1 | NM_016009.2 |
| SPHK1 | green | sphingosine kinase 1 | NM_021972.2 |
| TAX1BP1 | yellow | Tax1 (human T-cell leukemia virus type I) binding protein 1 | NM_006024.4 |
| GRIM19 | purple | cell death-regulatory protein GRIM19 | NM_015965.3 |
| TNFRSF10B | yellow | tumor necrosis factor receptor superfamily, member 10b | NM_147187.1 |
| DEDD2 | green | death effector domain containing 2 | NM_133328.2 |
| BCAP31 | pink | B-cell receptor-associated protein 31 | NM_005745.5 |
| NFKB1 | red | nuclear factor of kappa light polypeptide gene enhancer in B-cells 1 | NM_003998.2 |
| DSIPI | brown | delta sleep inducing peptide, immunoreactor | NM_004089.2 |
| YARS | brown | tyrosyl-tRNA synthetase | NM_003680.2 |
| C20orf97 | green | chromosome 20 open reading frame 97 | NM_021158.3 |
| CLU | green | clusterin | NM_001831.2 |
| INHBA | green | inhibin beta A | NM_002192.1 |
| BCL2L13 | green | BCL2-like 13 | NM_015367.2 |
| DAPK3 | brown | death-associated protein kinase 3 | NM_001348.1 |
| DFFA | brown | DNA fragmentation factor | NM_004401.1 |
| TP53BP2 | brown | tumor protein p53 binding protein 2 | NM_005426.1 |
| RTN4 | green | reticulon 4 | NM_007008.1 |
| BNIP3 | turquoise | BCL2/adenovirus E1B 19kDa interacting protein 3 | NM_004052.2 |
| PPP1R15A | brown | protein phosphatase 1 regulatory (inhibitor) subunit 15A | NM_014330.2 |
| GADD45A | brown | growth arrest and DNA-damage-inducible alpha | NM_001924.2 |
| GADD45B | brown | growth arrest and DNA-damage-inducible, beta | NM_015675.1 |
|  |  |  |  |
| Cell adhesion |  |  |  |
| ARHA | brown | ras homolog gene family, member A (ARHA) | NM_001664.1 |
| FN1 | brown | fibronectin 1 (FN1), transcript variant 1 | NM_002026.1 |
| PTPNS1 | green | protein tyrosine phosphatase, non-receptor type substrate 1 | NM_080792.1 |
| LAMA5 | yellow | laminin, alpha 5 | NM_005560.3 |
| CD44 | brown | CD44 antigen | NM_000610.2 |
| ARHE | green | ras homolog gene family, member E | NM_005168.2 |
| MICB | red | MHC class I polypeptide-related sequence B | NM_005931.2 |
| ITGB3BP | turquoise | integrin beta 3 binding protein (beta3-endonexin) | NM_014288.3 |
| PCDH1 | brown | protocadherin 1 | NM_002587.3 |
| ATP2A2 | blue | ATPase, Ca++ transporting, cardiac muscle, slow twitch 2 | NM_170665.2 |
| FLRT2 | blue | fibronectin leucine rich transmembrane protein 2 | NM_013231.2 |
| ADRM1 | blue | adhesion regulating molecule 1 | NM_175573.1 |
| IL8 | brown | interleukin 8 | NM_000584.2 |
| PLEKHC1 | green | pleckstrin homology domain containing, family C (with FERM domain) member 1 | NM_006832.1 |
| MKLN1 | green | muskelin 1, intracellular mediator containing kelch motifs (MKLN1). | NM_013255.2 |
| DLC1 | green | deleted in liver cancer 1 (DLC1), transcript variant 3. | NM_024767.2 |
| CYR61 | brown | cysteine-rich, angiogenic inducer, 61 (CYR61). | NM_001554.3 |
| RAC1 | greenyellow | ras-related C3 botulinum toxin substrate 1 | NM_006908.3 |
| GNE | red | glucosamine (UDP-N-acetyl)-2-epimerase/N-acetylmannosamine kinase | NM_005476.3 |
| LOXL2 | blue | lysyl oxidase-like 2 | NM_002318.1 |
| MICA | brown | MHC class I polypeptide-related sequence A | NM_000247.1 |
| COL4A1 | yellow | collagen, type IV, alpha 1 | NM_001845.3 |
| MFGE8 | yellow | milk fat globule-EGF factor 8 protein | NM_005928.1 |
| ERBB2IP | green | erbb2 interacting protein | NM_018695.1 |
|  |  |  |  |
| **Inflammatory response** |  |  |  |
| RAC1 | greenyellow | ras-related C3 botulinum toxin substrate 1 | NM_006908.3 |
| BCL6 | brown | B-cell CLL/lymphoma 6 | NM_001706.2 |
| MAIL | brown | molecule possessing ankyrin repeats induced by lipopolysaccharide | NM_031419.1 |
| IL8 | brown | interleukin 8 (IL8). | NM_000584.2 |
| LTA4H | black | leukotriene A4 hydrolase | NM_000895.1 |
| MIF | black | macrophage migration inhibitory factor | NM_002415.1 |
| HPSE | brown | heparanase | NM_006665.2 |
| NFKB1 | red | nuclear factor of kappa light polypeptide gene enhancer in B-cells 1 | NM_003998.2 |
| TPST1 | brown | tyrosylprotein sulfotransferase 1 (TPST1). | NM_003596.2 |
| CEBPB | green | CCAAT/enhancer binding protein (C/EBP), beta | NM_005194.2 |
| CXCL1 | brown | (melanoma growth stimulating activity, alpha) (CXCL1). | NM_001511.1 |
| LY96 | red | lymphocyte antigen 96 (LY96). | NM_015364.2 |
|  |  |  |  |
| Lipid metabolism |  |  |  |
| MGST1 | yellow | microsomal glutathione S-transferase 1 | NM_145792.1 |
| SPHK1 | green | sphingosine kinase 1 | NM_021972.2 |
| DCTN6 | yellow | dynactin 6 | NM_006571.2 |
| LOC221955 | green | KCCR13L | NM_139179.1 |
| ADM | brown | adrenomedullin | NM_001124.1 |
| OSBP | blue | oxysterol binding protein | NM_002556.2 |
| CYP51A1 | blue | cytochrome P450, family 51, subfamily A, polypeptide 1 | NM_000786.2 |
| LTA4H | black | leukotriene A4 hydrolase | NM_000895.1 |
| B4GALT5 | green | UDP-Gal:betaGlcNAc beta 1,4- galactosyltransferase, polypeptide 5 | NM_004776.2 |
| LTB4DH | yellow | leukotriene B4 12-hydroxydehydrogenase | NM_012212.2 |
| CLU | green | clusterin | NM_001831.2 |
| GNE | red | glucosamine (UDP-N-acetyl)-2-epimerase/N-acetylmannosamine kinase | NM_005476.3 |
| AKR1C3 | yellow | aldo-keto reductase family 1, member C3 | NM_003739.4 |
| CLN2 | yellow | ceroid-lipofuscinosis, neuronal 2 | NM_000391.2 |
|  |  |  |  |
| Protein folding |  |  |  |
| C12orf8 | yellow | chromosome 12 open reading frame 8 | NM_006817.2 |
| TCP1 | green | t-complex 1 | NM_030752.1 |
| BAG3 | green | BCL2-associated athanogene 3 | NM_004281.2 |
| FKBP4 | green | FK506 binding protein 4 | NM_002014.2 |
| HSPCB | green | heat shock 90kDa protein 1, beta | NM_007355.2 |
| HSPA8 | brown | heat shock 70kDa protein 8 | NM_153201.1 |
| DNAJB4 | green | DnaJ (Hsp40) homolog, subfamily B | NM_007034.3 |
| DNAJB11 | green | DnaJ (Hsp40) homolog, subfamily B, member 11 | NM_016306.3 |
| APG-1 | green | heat shock protein | NM_014278.2 |
| SIL1 | pink | endoplasmic reticulum chaperone | NM_022464.3 |
| DNAJA4 | green | DnaJ (Hsp40) homolog, subfamily A, member 4 | NM_018602.2 |
| DNAJB2 | green | DnaJ (Hsp40) homolog, subfamily B, member 2 | NM_006736.4 |
| TXNDC4 | red | thioredoxin domain containing 4 (endoplasmic reticulum) | XM_088476.6 |
| HKE2 | greenyellow | HLA class II region expressed gene KE2 | NM_014260.2 |
| HSPCA | green | heat shock 90kDa protein 1, alpha | NM_005348.2 |
| HSPD1 | green | heat shock 60kDa protein 1 | NM_002156.4 |
| DNAJA1 | green | DnaJ (Hsp40) homolog, subfamily A, member 1 | NM_001539.1 |
| HSPE1 | green | heat shock 10kDa protein 1 | NM_002157.1 |
| DNAJC7 | green | DnaJ (Hsp40) homolog, subfamily C, member 7 | NM_003315.1 |
| SDCCAG10 | black | serologically defined colon cancer antigen 10 | NM_005869.1 |
| CCT2 | green | chaperonin containing TCP1, subunit 2 | NM_006431.1 |
| CCT7 | yellow | chaperonin containing TCP1, subunit 7 | NM_006429.1 |
| DNAJB1 | green | DnaJ (Hsp40) homolog, subfamily B, member 1 | NM_006145.1 |
| DNAJA2 | blue | DnaJ (Hsp40) homolog, subfamily A, member 2 | NM_005880.2 |
| DNAJB9 | green | DnaJ (Hsp40) homolog, subfamily B, member 9 | NM_012328.1 |
|  |  |  |  |
| **Unfolded protein response** |  |  |  |
| XBP1 | green | X-box binding protein 1 | NM_005080.2 |
| UPF2 | pink | UPF2 regulator of nonsense transcripts homolog (yeast) (UPF2), transcript variant 2. | NM_015542.2 |
| HSPCB | green | heat shock 90kDa protein 1, beta | NM_007355.2 |
| HSPA8 | green | heat shock 70kDa protein 8 | NM_006597.3 |
| HSPA1A | green | heat shock 70kDa protein 1A | NM_005345.4 |
| HSPA1B | green | heat shock 70kDa protein 1B | NM_005346.3 |
| ATF4 | brown | activating transcription factor 4 | NM_182810.1 |
| DDIT3 | green | DNA-damage-inducible transcript 3 | NM_004083.3 |
| TXNDC4 | red | thioredoxin domain containing 4 | XM_088476.6 |
| SELS | yellow | selenoprotein S | NM_018445.4 |
| HSPB1 | green | heat shock 27kDa protein 1 | NM_001540.2 |
| HERPUD1 | green | homocysteine-inducible, endoplasmic reticulum stress-inducible, ubiquitin-like domain member 1 | NM_014685.1 |
| PPP1R15A | brown | protein phosphatase 1, regulatory (inhibitor) subunit 15A | NM_014330.2 |
|  |  |  |  |
| **Ubiquitin-dependent protein catabolism** |  |  |  |
| UBE2L3 | brown | ubiquitin-conjugating enzyme E2L 3 | NM_198157.1 |
| PSMB2 | pink | proteasome (prosome, macropain) subunit, beta type, 2 | NM_002794.3 |
| RBAF600 | brown | retinoblastoma-associated factor 600 | NM_020765.1 |
| PSMD14 | magenta | proteasome (prosome, macropain) 26S subunit, non-ATPase, 14 | NM_005805.2 |
| D13S106E | green | highly charged protein | NM_005800.3 |
| UBAP1 | brown | ubiquitin associated protein 1 | NM_016525.3 |
| TSG101 | yellow | tumor susceptibility gene 101 | NM_006292.2 |
| PSMB3 | purple | proteasome (prosome, macropain) subunit, beta type, 3 | NM_002795.2 |
| PSMB6 | purple | proteasome (prosome, macropain) subunit, beta type, 6 | NM_002798.1 |
| USP1 | magenta | ubiquitin specific protease 1 | NM_003368.3 |
| PSMB7 | purple | proteasome (prosome, macropain) subunit, beta type, 7 | NM_002799.2 |
| PSMB4 | greenyellow | proteasome (prosome, macropain) subunit, beta type, 4 | NM_002796.2 |
| USP14 | yellow | ubiquitin specific protease 14 | NM_005151.2 |
| SQSTM1 | brown | sequestosome 1 | NM_003900.2 |
| USP22 | yellow | ubiquitin specific protease 22 | XM_042698.6 |
|  |  |  |  |
| **Immunse response** |  |  |  |
| IFITM3 | purple | interferon induced transmembrane protein 3 | NM_021034.1 |
| MAIL | brown | molecule possessing ankyrin repeats induced by lipopolysaccharide | NM_031419.1 |
| XBP1 | green | X-box binding protein 1 | NM_005080.2 |
| FN1 | brown | fibronectin 1 | NM_002026.1 |
| HLA-C | turquoise | major histocompatibility complex, class I, C | NM_002117.3 |
| HPSE | brown | heparanase | NM_006665.2 |
| ULBP2 | green | UL16 binding protein 2 | NM_025217.2 |
| BCL6 | brown | B-cell CLL/lymphoma 6 | NM_001706.2 |
| TPST1 | brown | tyrosylprotein sulfotransferase 1 | NM_003596.2 |
| IL11 | brown | interleukin 11 | NM_000641.2 |
| HSPA1B | green | heat shock 70kDa protein 1B | NM_005346.3 |
| MICB | red | MHC class I polypeptide-related sequence B | NM_005931.2 |
| IL8 | brown | interleukin 8 | NM_000584.2 |
| CEBPB | green | CCAAT/enhancer binding protein (C/EBP), beta | NM_005194.2 |
| BCAP31 | pink | B-cell receptor-associated protein 31 | NM_005745.5 |
| LY96 | red | lymphocyte antigen 96 | NM_015364.2 |
| FCGRT | brown | Fc fragment of IgG, receptor, transporter, alpha | NM_004107.3 |
| NFKB1 | red | nuclear factor of kappa light polypeptide gene enhancer in B-cells 1 | NM_003998.2 |
| D2S448 | tan | Melanoma associated gene | XM_056455.3 |
| RAC1 | greenyellow | ras-related C3 botulinum toxin substrate 1 | NM_006908.3 |
| DXYS155E | green | DNA segment on chromosome X and Y (unique) 155 expressed sequence | NM_005088.2 |
| CLU | green | clusterin | NM_001831.2 |
| FTH1 | black | ferritin, heavy polypeptide 1 | NM_002032.1 |
| CXCL1 | brown | chemokine (C-X-C motif) ligand 1 | NM_001511.1 |
| INHBA | green | inhibin, beta A | NM_002192.1 |
| LTA4H | black | leukotriene A4 hydrolase | NM_000895.1 |
| MIF | black | macrophage migration inhibitory factor (glycosylation-inhibiting factor) | NM_002415.1 |
| MICA | brown | MHC class I polypeptide-related sequence A | NM_000247.1 |
| NFIL3 | brown | nuclear factor, interleukin 3 regulated | NM_005384.1 |
| CD2BP2 | tan | CD2 antigen (cytoplasmic tail) binding protein 2 | NM_006110.1 |
| HTGN29 | red | HTGN29 protein | NM_020199.1 |

The genes which were synergistically coregulated by DEP and ox-PAPC in the gene network were submitted to pathway analysis with EASE program in Go Biological Process platform. The table lists genes belonging to selected pathways (EpRE regulated genes, Apoptosis, Cell adhesion, Inflammatory response, Lipid Metabolism, Protein folding, Unfolded Protein Response, Ubiquitin-dependent protein catabolism and Immune response), module allocation and accession number.
