# Supplementary material for: Air-pollutant chemicals and oxidized lipids exhibit genome-wide synergistic effects on endothelial cells
Source: Genome Biol. 2007 Jul 26;8(7):R149. doi: 10.1186/gb-2007-8-7-r149 (PMC2323217; doi:10.1186/gb-2007-8-7-r149)
Supplement: Additional data file 6 — SIs of representative genes as determined from microarray and qPCR data. [file gb-2007-8-7-r149-S6.doc]

**Additional data file 6**. Synergistic indexes

**Microarray analysis**

| Condition | IL8 | Hmox1 | SELS | HSPA8 | XBP1 | ATF4 | ATF3 | DUSP1 | CXCL1 | PDLIM1 | IL11 | SOD1 | NQO1 |
| --- | --- | --- | --- | --- | --- | --- | --- | --- | --- | --- | --- | --- | --- |
| DEP5+ox10 | 1.3* | 1.1* | 1.5* | 1.2* | 2.2 |  |  | 0.1 | 0.5 | 0.4 | 0.7 |  | 1.3* |
| DEP5+ox20 | 2.7* | 0.9 | 0.9 | 1.3* | 2.0* | 3.3* | 1.4 | 0.3 | 1.3* | 0.7 | 0.7 | 1.6* | 0.8 |
| DEP5+ox40 | 1.1 | 0.8 | 0.6 | 1.4* | 1.6* | 1.0 | 4.0* | 1.5* | 1.8* | 1.2* | 1.6* | 0.9 | 0.4 |

**qPCR analysis**

| Condition | IL8 | Hmox1 | SELS | HSPA8 | XBP1 | ATF4 | ATF3 | DUSP1 | CXCL1 | PDLIM1 | IL11 |
| --- | --- | --- | --- | --- | --- | --- | --- | --- | --- | --- | --- |
| DEP5+ox10 | 1.1 | 1.8* | 1.2* | 1.4* | 2.1* | 1.7* | 3.9 | 1.3 |  | 2.2 | 1.8* |
| DEP5+ox20 | 8.7* | 1.3* | 1.2* | 1.4* | 2.3* | 1.8* | 12* | 4.0 | 7.7* | 1.3 | 2.0* |
| DEP5+ox40 | 6* | 0.8 | 1.5* | 1.1 | 2.1* | 1.4* | 6.1* | 9.6* | 3.1* | 5.4* | 5.1* |

Synergistic indexes of representative genes as determined from microarray (top) and qPCR (bottom) data. *: presence of synergy as defined in the methods. Microarray and qPCR analysis were conducted on triplicate samples from independent experiments. Microarray data was also validated by qPCR within the same experiment (data not shown). DEP5 + (ox10, ox20, ox40): DEP 5 μg/ml + ox-PAPC 10, 20 and 40 μg/ml respectively.  : < 0.1
