# Supplementary material for: Air-pollutant chemicals and oxidized lipids exhibit genome-wide synergistic effects on endothelial cells
Source: Genome Biol. 2007 Jul 26;8(7):R149. doi: 10.1186/gb-2007-8-7-r149 (PMC2323217; doi:10.1186/gb-2007-8-7-r149)
Supplement: Additional data file 7 — The recovery of major organic fractions from 1 g DEP. [file gb-2007-8-7-r149-S7.doc]

**Additional data file 7.** Recovery of major organic fractions from 1 g of DEP

| Fraction | Elution Solvent | Amount (mg) | Recovery (%)^a^ |
| --- | --- | --- | --- |
| Aliphatic | Hexane | 235 | 23.5 |
| Aromatic | Hexane/methylene chloride (3/2, v/v) | 105 | 10.5 |
| Polar | Methylene chloride/methanol (1/1, v/v) | 100 | 10 |
| Total |  | 440 | 44 |

^a^ The amount of asphaltene from 1 g of DEP = 289.7 mg, which represents 29% of particle mass. This data has been published in the Journal of Immunology [58] and Copyright 2004 The American Association of Immunologists, Inc.
