# Supplementary material for: Air-pollutant chemicals and oxidized lipids exhibit genome-wide synergistic effects on endothelial cells
Source: Genome Biol. 2007 Jul 26;8(7):R149. doi: 10.1186/gb-2007-8-7-r149 (PMC2323217; doi:10.1186/gb-2007-8-7-r149)
Supplement: Additional data file 8 — The content of polycyclic aromatic hydrocarbons in crude DEP extract and fractions. [file gb-2007-8-7-r149-S8.doc]

**Additional data file 8.** PAH content in each DEP fraction^a^

| PAH | PAH Content (µg/g DEP) | | | |
| --- | --- | --- | --- | --- |
|  |  |  |  |  |
|  |  |  |  |  |
|  | Crude extract | Aliphatic | Aromatic | Polar |
| NAP | 89 | 0.04 | 3.71 | 0 |
| ACE | 67 | 0 | 0.48 | 0 |
| FLU | 153 | 0 | 6.18 | 0 |
| PHE | 1576 | 0.04 | 110.87 | 0 |
| ANT | 24 | 0 | 0.95 | 0.12 |
| FLT | 678 | 0 | 45.13 | 1.01 |
| PYR | 530 | 0 | 23.37 | 0.05 |
| BAA | 91 | 0 | 6.18 | 0 |
| CRY | 158 | 0 | 7.7 | 0 |
| BBF | 48 | 0 | 1.71 | 0 |
| BKF | 20 | 0 | 0.29 | 0 |
| BAP | 16 | 0 | 0 | 0 |
| DBA | 16 | 0 | 0.1 | 0 |
| BGP | 18 | 0 | 0.1 | 0 |
| IND | 18 | 0 | 0.1 | 0 |

^a^ PAH content was measured as previously described [66]. Sixteen standard PAH were used to quantitate the PAH content in each fraction. NAP, naphthalene; ACE, acenaphthalene; FLU, fluorene; PHE, phenanthrene; ANT, anthracene; FLT, fluoranthene; PYR, pyrene; BAA, benzo(a)anthracene; CRY, chrysene; BBF, benzo(b)fluoranthene; BKF, benzo(k)fluoranthene; BAP, benzo(a)pyrene; DBA, dibenz(a,h)anthracene; BGP, benzo(ghi)perylene; IND, indeno(1,2,3,-cd)pyrene. This data has been published in the Journal of Immunology [58] and Copyright 2004 The American Association of Immunologists, Inc.
