# Supplementary material for: Air-pollutant chemicals and oxidized lipids exhibit genome-wide synergistic effects on endothelial cells
Source: Genome Biol. 2007 Jul 26;8(7):R149. doi: 10.1186/gb-2007-8-7-r149 (PMC2323217; doi:10.1186/gb-2007-8-7-r149)
Supplement: Additional data file 9 — The quinone content in crude DEP extract and fractions. [file gb-2007-8-7-r149-S9.doc]

**Additional data file 9.** Quinone content in DEP fractions^a^

| Quinones | Quinone Content in DEP Fractions (µg/g DEP) | | | |
| --- | --- | --- | --- | --- |
|  |  |  |  |  |
|  |  |  |  |  |
|  | Crude extract | Aliphatic | Aromatic | Polar |
| 1,2 NQ | 22.34 | ND | ND | 2.28 |
| 1,4 NQ | 19.94 | ND | ND | 6.91 |
| 9,10 PQ | 18.73 | ND | ND | 6.03 |
| 9, 10 AQ | 69.34 | ND | ND | 36.86 |

^a^ Quinone contents in crude DEP extract and fractions were analyzed as described in Materials and Methods. Four standard quinones were used for quantitation: 1,2 NQ, 1,2-naphthoquinone; 1,4 NQ, 1,4-naphthoquinone; 9,10 PQ, 9,10-phenanthrenequinone; and 9,10 AQ, 9,10-athraquinone. ND, not detected. This data has been published in the Journal of Immunology [58] and Copyright 2004 The American Association of Immunologists, Inc.

.
